# Kangaroo mother care: using formative research to design an acceptable community intervention

**DOI:** 10.1186/s12889-018-5197-z

**Published:** 2018-03-02

**Authors:** Sarmila Mazumder, Ravi Prakash Upadhyay, Zelee Hill, Sunita Taneja, Brinda Dube, Jasmine Kaur, Medha Shekhar, Runa Ghosh, Shruti Bisht, Jose Carlos Martines, Rajiv Bahl, Halvor Sommerfelt, Nita Bhandari

**Affiliations:** 1 Centre for Health Research and Development, Society for Applied Studies, 45, Kalu Sarai, New Delhi, 110016 India; 2 Institute for Global Health, Faculty of Population Health Sciences, University College of London, London, UK; 3 Centre for Intervention Science in Maternal and Child Health, Centre for International Health, University of Bergen, Bergen, Norway; 4 Department of Maternal, Newborn, Child and Adolescent Health, World Health Organization, Geneva, Switzerland

**Keywords:** Kangaroo mother care, Household trials, Formative research

## Abstract

**Background:**

Low and middle income countries (LMICs), including India, contribute to a major proportion of low birth weight (LBW) infants globally. These infants require special care. Kangaroo Mother Care (KMC) in hospitals is a cost effective and efficacious intervention. In institutional deliveries, the duration of facility stay is often short. In LMICs, a substantial proportion of deliveries still occur at home and access to health care services is limited. In these circumstances, a pragmatic choice may be to initiate KMC at home for LBW babies. However, evidence is lacking on benefits of community-initiated KMC (cKMC). Promoting KMC at home without an understanding of its acceptability may lead to limited success.

**Methods:**

We conducted formative research to assess the feasibility, acceptability and adoption of cKMC with the aim of designing an intervention package for a randomised controlled trial in LBW infants in Haryana, India. Qualitative methods included 40 in-depth interviews with recently delivered women and 6 focus group discussions, two each with fathers and grandfathers, grandmothers, and community health workers. A prototype intervention package to promote cKMC was developed and tested in 28 mother-infant pairs (of them, one mother had twins), using Household (HH) trials.

**Results:**

We found that most mothers in the community recognized that babies born small required special care. In spite of not being aware of the practice of KMC, respondents felt that creating awareness of KMC benefits will promote practice. They expressed concerns about doing KMC for long periods because mothers needed rest after delivery. However, the cultural practice of recently delivered women not expected to be doing household chores and availability of other family members were identified as enablers. HH trials provided an opportunity to test the intervention package and showed high acceptability for KMC. Most mothers perceived benefits such as weight gain and increased activity in the infant.

**Conclusions:**

Community-initiated KMC is acceptable by mothers and adoption rates are high. Formative research is essential for developing a strategy for delivery of an intervention.

**Trial registration:**

Trial registration number CTRI/2015/10/006267.

Name of Registry: Clinical Trials Registry - India.

URL of Registry: http://ctri.nic.in/Clinicaltrials/login.php

Date of Registration: 15/10/2015.

Date of enrolment of the first participant to the trial: 18/04/2015.

## Key messages

1. Community-initiated KMC - acceptable by mothers; adoption rates are high
2. Formative research essential for developing a strategy for delivery of an intervention
3. Household trials are a useful way to test acceptability of KMC

## Background

Every year, around 15 million infants weighing less than 2500 g are born, most of them in low and middle income countries [1]. These low birth weight (LBW) infants are at increased risk of growth retardation, infections, developmental delay and death during infancy and childhood [2–5]. Many of the families in whom these LBW infants are born do not have access to or cannot afford the cost of conventional neonatal care that includes the use of incubators and skilled personnel [6–8]. Kangaroo Mother Care (KMC) was developed over 30 years ago in response to constraints of access and affordability, and has subsequently been shown to have benefits over incubator care. The key features of KMC are early, continuous and prolonged skin-to-skin contact (SSC) between the mother and the baby and exclusive breastfeeding [9].

KMC provided to stable babies in hospitals is associated with a 40% relative reduction in the risk of death, 65% reduction in the risk of nosocomial infections, and a 72% reduction in hypothermia, at discharge or 40–41 weeks postmenstrual age compared to conventional care [10, 11]. Other benefits include improvement in weight and length, cognitive development, mother-child bonding, maternal confidence in caring for their babies and reduced stress in mothers and babies [10, 12]. However, the evidence of benefits is largely from research studies in hospitals with KMC initiated by skilled health workers [10] and evidence is lacking on whether KMC initiated at home has similar benefits.

In many LMICs, the proportion of pregnant women delivering in facilities are increasing but mothers are discharged early and ensuring continuation of facility-initiated KMC at home after discharge is therefore, a priority. Many LBW babies, particularly from poor families, are born at home [13, 14]. For such babies, cKMC is an important intervention. A large study in Bangladesh, designed to test the mortality impact of community-based KMC, provided useful insights into the barriers in implementing KMC but evidence on impact was not conclusive [15]. Literature on barriers and facilitators to implementing KMC at home is scanty [16–18]. Without an understanding of these, initiating and promoting KMC at home may meet with limited success.

In preparation for a randomized controlled trial (RCT) to ascertain the mortality impact of cKMC in LBW babies, we conducted formative research from December 2014 to June 2015. The aim of this work was to assess the feasibility and acceptability of cKMC, to understand the barriers and facilitators to the practice, and based on the findings, design an intervention package and delivery strategy that would achieve high adoption rates among caregivers of LBW infants.

## Methods

### Conceptual framework considered for achieving high uptake of KMC

**Fig. 1:**
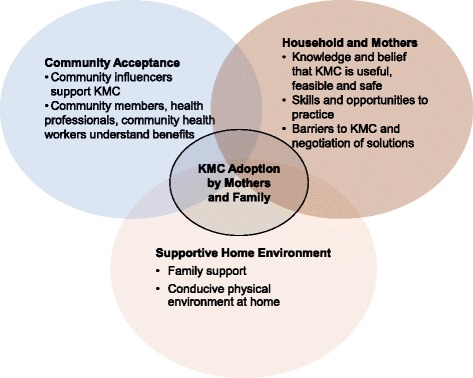
Conceptual Model to Achieve High Adoption of KMC

A conceptual framework was developed to guide the formative research and identify factors important for promoting KMC in the community (Fig. 1). We believed that the adoption of KMC by the mother and family members would be largely influenced by factors at various levels: individual, household, community, social, cultural and religious. Endorsement by health care providers and community workers would also be important. Awareness about the vulnerability of LBW babies, the need for special care, the knowledge about benefits of KMC, the skills to practice it including being able to resolve problems when they occurred, and a supportive home environment were some hypothesised pathways embedded in the framework.

### Study setting and population characteristics

The study setting for the planned RCT was rural and semi-urban areas of two districts, Faridabad and Palwal, in the state of Haryana, India, covering a population of about 1.4 million. For the formative research, one rural and semi-urban area from the two districts covering a population of about 200,000 was identified. The population was representative of the two districts in terms of family income and ethnicities. A fourth of babies weighed less than 2500 g at birth [19]. The study was conducted between December 2014 to June 2015.

### Study team

The study team comprised of anthropologists, social scientists, physicians and health workers equivalent to the government Auxiliary Nurse Midwives (ANMs; key health workers who form the interface between community and the public health services; (http://nhm.gov.in/; http://health.bih.nic.in/Docs/Guidelines/Guidelines-Sub-Centers-(Revised)-2012.pdf) and Accredited Social Health Activists (ASHAs, the community level health workers, for home visits and referral for reproductive and child health services, http://nhm.gov.in/communitisation/asha/about-asha.html). Prior to initiation of community activities, the team visited the KMC ward of a premier tertiary care hospital and received training on KMC by the neonatologists who also made them practice the technique. Methods for formative research were finalized in a workshop held with study advisors (JM, RB, HS and ZH) and national experts. An international expert (ZH) trained the team in data collection and analysis.

### Data collection

Forty in-depth interviews (IDIs) were conducted with mothers of LBW babies within 10 days of birth. Information on births was sought from government ASHAs, traditional birth attendants (TBAs) and private health care providers practicing in the area.

Two focus group discussions (FGDs) were conducted with mothers and grandmothers of children aged less than 2 years. Two FGDs were also conducted with males – fathers and grand fathers. Each FGD had 8–9 participants. The topics covered were delivery and newborn caring practices, and knowledge and practices pertaining to babies born LBW.

Photographs of women practicing KMC were shown to participants and their reactions were observed. As no one recognized the practice, KMC was explained and feedback was taken on what the concerns would be if mothers were asked to practice KMC. The information collected was reviewed daily to identify gaps and additional data was collected, whenever necessary.

Two FGDs covering the same topics were also done with community workers (ASHAs and ANMs) working in the study area.

### Household trials and developing a prototype intervention

Based on initial findings of the formative research, a prototype intervention package was developed. The package included promotion of SSC for as long as possible during day and night, practiced in a position that the mother was comfortable (sitting, semi-reclining or supine with head raised); and promotion of optimal breastfeeding practices. These included early initiation of breastfeeding, feeding colostrum, not giving prelacteal feeds, feeding the baby on demand or every 2 hourly, at least 8 times in 24 h, and exclusive breastfeeding. Binders to hold the baby in KMC position designed by the study team and disposable diapers bought from the market were also offered. The intent of offering the latter was that we felt that home-made cloth diapers used in these communities would need to be changed whenever they got wet and this would necessitate removing the baby from the KMC position.

During household trials, the mother was visited daily in the first week and thereafter on alternate days. Frequent visits were made to understand whether KMC could be initiated at home, the duration of SSC in hours per day and number of days mothers were willing to practice, the number of days they actually practiced, perceived benefits, the problems encountered and possible ways to resolve them with the overall aim of achieving high adoption rates.

### Procedures for household trials

Babies weighing less than 2500 g were identified within 7 days of birth. Twenty-eight mother-infant pairs were identified; one mother had twin babies. We attempted to cover diversity in terms of rural and semi urban residence, young primipara and older multipara, religion and place of delivery (hospital and home). At the first visit, counselling was done by higher level worker (the study ANM). The study ANM in the presence of the study ASHA helped the mother to initiate KMC at home, explained the technique and benefits to the mother and other family members and helped the mother put the baby in the KMC position. The mother was advised to practice it for as many hours as possible throughout day and night till the baby was aged 28 days or wriggled out; the latter indicating the baby did not want to stay in that position. Binders and disposable diapers were offered.

The subsequent home visits were made by the study ASHA; ANMs were called only in case of problems.

Home visits were made daily during the initial week and subsequently on alternate days till the baby was aged 28 days. During this visit, the mother was asked to share her experiences. A counselling guide listing problems reported and their resolution by the study team was developed and updated. The common barriers reported and solutions accepted by mothers are summarized in Table 1.

**Table 1:** Barriers and Feasible Solutions

Barriers Reported	Feasible Solution(s)
For exclusive breastfeeding	
Baby too weak to suck	Mothers counseled and demonstrated with hands on practice on feeding expressed breast milk
Colostrum discarded; pre-lacteal feeds given; feel that baby needs water during summer	Counseled about benefits of feeding colostrum; exclusive breastfeeding and disadvantages of pre-lacteal feeding. Also counseled about adequacy of water content for the baby in breast milk
Scanty milk flow soon after birth; inadequacy of breast milk, feels that baby should be given top milk	Explained to mothers that baby does not need milk in large volumes during the initial few days after birth and that frequent breastfeeding increases milk flow
Inverted, small or cracked nipples; engorged breasts, breast abscess	Demonstration on use of syringe to pull out nipple; feed expressed breast milk till cracked nipple heals; mothers taught to practice hot fomentation and express breast milk; facilitated referral for treatment of breast abscess
No milk secretion, mother severely ill	Lactation support provided. If mother severely ill, option of breast feeding by wet nurse suggested to the family. If not available, top milk advised with appropriate hygiene and preparation methods
For continued skin-to-skin contact	
Heat and humidity, especially during summers	Family counseled to use fan or cooler without exposing baby directly or use hand fans during power cuts. Water sprinkled on floor, floor mopped with cold water to reduce room temperature
Lack of privacy	For single room homes, partition with cloth, plastic curtain or a vertically placed jute cot was used for privacy. In households with more than one room, mother moved to a separate room.
Abdominal pain post delivery, postpartum fatigue, mother ill or too weak	Mother assisted to sit comfortably using pillow or soft clothes and change positions often (semi-reclining, supine) or give KMC while moving around. Family members counseled to help mother and to give adequate food to the mother. Mothers referred to health facility if unwell. If other family members not available, encouraged to call relatives or neighbours to help.
Reluctance to wear front open clothes either due to cultural reasons or shyness	Long binders similar to shirts worn in the setting that cover the mother’s body, designed. Alternatively, mothers advised to wear husband’s shirts or night gown.
Heat from mother’s abdomen gets transmitted to baby causing diarrhea or vomiting, concern that baby’s stomach would get pressed and cause vomiting	Counseled that heat from mother’s body is essential for a LBW baby who is unable to maintain own body temperature. Baby feels comfortable and secure when placed on mother’s body and the stomach does not get pressed in this position
Concern regarding possibility of injuring umbilical stump and bleeding in SSC due to friction with mother’s skin	Family counseled and reassured that SSC position does not cause friction or injury to umbilical stump.
Fear of neck deformity in baby if kept with head turned on one side, in the same position for long; difficulty in placing baby in SSC due to lack of neck control	Counseled that KMC position, if followed correctly, does not cause deformity; and baby’s neck is well supported and rests on mother’s breasts;
SSC would be interrupted as the baby needs to be removed from SSC frequently to clean stool	Mothers demonstrated on how to clean the baby without removing from SSC position
Concern that baby would not allow the mother to resume routine work after the period of rest is over due to excessive attachment	Counseled about the importance of doing KMC and its long term effect on baby’s growth and development. Also that KMC promotes healthy bonding rather than dependency.
Fear that mother’s infections could pass on to the baby through sweat	Family counseled and assured that infections are not transmitted through sweat
Refusal to undress the baby for KMC in winter; feared that the baby will catch cold	Family advised to keep doors and windows shut and use room heater, whenever possible. Mother demonstrated on how to undress baby after placing in SSC position; use of sleeveless front open woolen sweaters promoted for the baby in families who insisted on woolen clothing
Apprehensions about giving SSC at night: fear of suffocating or smothering the baby if she falls asleep and turns to her side crushing the baby	Demonstrated how to lie down with support on either side with pillows or blankets, or rolled up old clothes (if pillows not available) to prevent her from turning on her side. This way they could avoid smothering the baby while asleep
Inability to practice SSC while moving around	Mother encouraged to use binders
Refusal to use tube top elastic binders: fear of suffocating the baby	Mothers demonstrated on placing baby in SSC in regular clothes. Tube tops given to women who felt comfortable
Difficulty in doing KMC for twins	Mother assisted in placing both babies in SSC simultaneously; family members requested for help
Time	
Lack of time due to household responsibilities	Mother-in-law and other family members counseled and encouraged to share household chores.
Limited family support, particularly in nuclear families	Mother encouraged to call relatives or neighbors or practice KMC for longer hours at night

### Data analysis

Data was analysed on an ongoing basis in NVivo (version 11.0) using thematic analysis. Anonymized data were analyzed in the local language so the researchers stayed as close to the participant’s verbatim as possible. Each interview with the respondent was recorded with prior written informed consent. These interviews were expanded and transcribed. The transcriptions were reviewed by the study co-ordinators, these were read carefully to understand and identify the emerging themes. Provisional categories were developed and refined after group reviews and a final set of key themes and sub themes were identified. Excerpts from the original transcripts were included in the analysis.

A bottom up analytical approach was used to understand the experiences and perspectives of the participants by coding and categorizing their stories and responses under identified themes and sub themes. The codes were grouped on the same topic into themes and sub themes. The total number of times a theme appeared and the total number of respondents with the theme were analysed. A matrix of framework analysis was prepared to display the themes, sub themes and respondent characteristics (case and theme based analysis).

## Results

### Characteristics of the participants

The mean age of mothers was 23.2 (SD 3.5) years. A third of mothers had never been to school, four-fifths lived in extended families (an extension of nuclear family to include grandparents and other relatives). Around 15% were Muslims and the remaining Hindus.

The mean (SD) age (in years) of grandmother, grandfathers and fathers was 52.5 (SD 10.7), 57 (SD 11.6) and 25 (SD 5.6) respectively.

### Findings

As the findings from mothers and grandmothers were similar, these are presented together. Similarly, the findings from fathers and grandfathers were mostly identical and are therefore, reported together. Findings from interactions with community health workers are presented separately.

#### Perception of babies born small or before time and need for special care

When asked about characteristics of babies who are born small or before time, and whether it is possible to differentiate these babies from normal babies, a tenth of the mothers and grandmothers mentioned that birth weights are used to categorize babies as normal or small. About two-fifth mentioned that babies born small are weak. Other characteristics mentioned were that these babies are small in size (60%), feel lighter (*halka;* 52%), are pale (30%), look like old persons with wrinkled skin (25%) and do not cry at birth (3 respondents). However, they were not aware of what the birth weight of a normal baby should be and said that they only became aware of a problem in the baby with regard to weight when informed by doctor, nurse or ASHA (http://nhm.gov.in/communitisation/asha/about-asha.html).

Around three-fourth of mothers and grandmothers said that they were aware that some babies were born ‘before time’ or ‘*samay se pehle’*. The characteristics of such babies are that they are weak (*kamjor*) compared to babies born timely, unable to feed (*doodh nahi pe pate*), look unwell (*dekhne me bimar*), movements are less than that of normal babies (*samanya baccho ke mukabale mein kam hilte-dulte hain)*, they have thin arms and legs (*patle haath aur pair)* and small face and eyes (*chhota chehra aur aankhe*), feel like jelly (*gilgila hote hai*) and have less blood (*khoon ki kami).*

“*Such babies sleep most of the time, do not wake up even if they are hungry, and cry very less. They are very fragile and therefore require to be treated in a special way, even at home”* (**Mother)**

Three-fourths of mothers and grandmothers said that babies born small or before time need special care because they are weak, at higher risk of falling ill frequently and therefore need an incubator. *“Babies that are born weak need special care. They are usually kept in glass (sheesha) for 10-15 days. After they are out of glass, they should be protected from cold, bathed less frequently, wrapped in cotton or clothes,* given ‘*ghutti’ (*locally-prepared herbal digestive liquid) *to make them strong and breastfed frequently. Since they are unable to suck breast milk, buffalo milk or dibbe wala doodh (powdered milk) should be given”* (**Grandmother**)

Quite unlike mothers and grandmothers, nearly four-fifths of the fathers and grandfathers were confused and unaware of birth weight or being born before time as being matters of concern. When asked what should be done if babies are born small, three-fourths of them said that such babies require special care, such as formula milk (*upar ka doodh*) or buffalo milk in addition to breast milk and oil massage to strengthen their bones. A few mentioned locally-prepared herbal digestive liquid for newborns (*ghutti)* as being necessary to aid digestion; around half suggested that a natural product extracted from deer (kasturi *ki goli*) needed to be given in winter to keep them warm.

#### Reactions to photographs showing mothers practicing KMC

Most mothers and grandmothers were curious. Over half described the photographs as showing babies being put to sleep and the mother showing love to her baby. Three mothers out of the 40 said that baby was tied with cloth to prevent the baby from falling. Two mentioned that the babies were being provided with the mothers’ warmth.

*“The baby is kept close to the heart. He is covered with clothes so that he remains warm. The baby is sleeping and after breastfeeding, babies usually sleep comfortably in this position”* (**Mother**)

A fourth of the mothers and grandmothers however, felt it was not a good practice to keep baby on the chest because babies would get used to mothers’ smell and closeness and not leave the mother later when she has to do household chores after the initial period of rest. Only a tenth of the mothers and grandmothers had heard about SSC, either from a health worker or hospital and they described it as placing the baby on mother’s chest because it helps in starting breast feeding, reduces need of incubator and promotes weight gain. However, none of them mentioned that it is for small babies or babies born before time. Only one mother had practiced SSC for her older child. She was advised to do so by a doctor from a primary health centre (http://nhm.gov.in/nrhm-components/health-systems-strengthening/infrastructure.html) to give SSC for 1 to 2 h each day after her baby was born. She had therefore practiced for 1 month, for an hour or two each day.

Only two fathers and grandfathers responded that the baby will receive mother’s warmth and protection from cold with KMC and that it would lead to increased weight gain.

#### Views on whether it would be possible for mothers to practice KMC at home

Nearly all mothers and grandmothers said mothers will practice KMC at home in spite of any difficulties that they encounter. Some concerns reported were profuse sweating in mother leading to skin rash in the baby during summer when heat and humidity were extreme and lower back ache because of remaining in one position for long and inability to do household chores (Table 1). Placing rolled up clothes, blankets and pillows as back support rather than insisting that mothers place the bed against the wall for back support was easily accepted because of the belief that if placed against the wall, evil spirits would enter the mother’s body. They however, said that KMC was only possible to do after 7 to 10 days of age as the cord falls off by that time and only then the baby can be placed on the mother’s chest. The concern for starting KMC before the cord falls is that it could rub against the mother’s skin, while in KMC position, and this could lead to bleeding in the cord stump.

Four-fifths of the mothers said round-the-clock KMC is not possible as mothers are weak post delivery, have backache and require rest especially at night. Also, babies may be smothered if the mother falls asleep. However, a longer duration was possible to give if grandmothers and other family members helped the mother in doing KMC and this would be possible in extended families. Most grandmothers said that the mother can do KMC for half hour to one-and-a half hours at a stretch, 3 to 4 times a day.

*“The mother can keep the baby in this position for one-and-half hours only, she cannot sit for long. A longer period for more than 2 hours at a stretch is possible only while lying down. She can do like this for 3-4 times a day. If mother falls asleep, the baby will be smothered”* (**Grandmother**)

However, all grandmothers agreed that since mothers do not do any household chores for some time after delivery, practising KMC should be possible at least during this time.

When disposable diapers were offered to the mother to use instead of home-made ones as there may be leakage from the latter if the baby passed urine or stools in the KMC position, most mothers and grandmothers were reluctant to use these for babies. They feared that use of thick diapers would result in skin rash, skin peeling, and the edge of diaper may hurt the umbilical stump. Besides, disposable diapers were believed to be unhygienic as they trapped urine and stool for long periods. Also, because of their thickness, they may increase the gap between the legs, leading to future deformity. Only two mothers mentioned the benefits of using disposable diapers i.e. they absorbed urine without feeling wet. Almost half the mothers reported using old folded cloth in triangular shape as diapers. The passing of urine by the baby while in the KMC position was not perceived to be a problem as the volume was less and would dry off quickly. The mother would need to clean herself only after the baby passed stools.

The views of fathers and grandfathers on whether mothers would be able to practice KMC at home was positive; they too felt that mothers get the 40-days period of rest after delivery and during this time it would be possible to practice KMC.

#### Availability of family support for practising KMC at home

When asked whether other family members could also help with KMC, about three-fourths of the mothers said that other family members, including fathers could help. Mothers would first do KMC themselves and when tired ask other family members. Grandmothers said they would be willing to help as it was beneficial for their grand children. Both mothers and grandmothers agreed that males (fathers or grandfathers) will not want to do KMC.

*“The male members work outside home and return late at night and are tired. They will not like the smell of urine and stool of the baby but some fathers may keep the baby on their chest before sleeping”* (**Mother**)

A few mothers said that since child care is the responsibility of the mother alone, no one in the family would help; however, the father may agree to help for a male baby. Mothers and grandmothers were unsure whether mothers in urban areas would be able to practice KMC as most families in the urban areas are nuclear with no additional family members. Mothers in nuclear families had to resume household chores soon after delivery, as early as the next day. In such cases, they suggested that relatives could be called to help.

When fathers and grandfathers were asked whether they would help in doing KMC, the majority said that they would be too scared to handle such small babies. Also, in rural areas, men do not like the idea of placing the baby against their chest. Two fathers said they would keep the baby in the KMC position but only for a few hours and that too while the baby was asleep after a feed, or at night. They would do this so that their wives would get some time to sleep.

#### Views of community health workers (ASHAs and ANMs)

As ASHAs and ANMs have deep understanding of the community, in addition to knowledge about KMC and its benefits, their views on practicing KMC at home, anticipated barriers and enablers were explored.

All ASHAs and ANMs were aware of KMC and its benefits for LBW and preterm babies. They said that KMC could be practiced at home at least for the initial 1 to 1.5 months post delivery, when mothers in most homes are not required to do household work. However, the desirable duration of KMC reported by the health workers was an hour each time and 3 times a day (morning, afternoon and night). Few ASHAs and ANMs mentioned that mothers-in-law may disapprove as the baby would get used to mother’s smell if kept close, making it difficult for the mother to resume household responsibilities later.

Other anticipated difficulties reported were lack of family support, non-conducive home environment such as lack of availability of a separate chair or bed to do KMC, limited privacy in extended families, the heat and humidity in summer, backache, painful episiotomy stitches and not being able to wear front open clothes due to cultural reasons. They also suggested that mothers should be taught KMC in the hospital post delivery before discharge.

*“Mothers don’t sit on chairs; elders, male members, children and guests have right to chairs. Women are on the floor or jute cot (charpai). With everyone around, mother will be reluctant to undress. KMC in summer will not be possible because of the heat and frequent power cuts.”* **(ANM)**

Quite unlike the mothers and grandmothers, the health workers believed that disposable diapers were necessary and that all families would like to use them except those who could not afford to buy them.

#### Recognition of KMC

Kangaroos were unknown in the community, except among health workers. Local names best describing the practice, were explored during IDIs and FGDs. Common suggestions were ‘*chaati se chipkana’ (*to stick to the chest); “*pyaar se lena*” (to hold with love); ‘*chaati se lagaana’* (to place on the chest); *‘maa ki mamta*’ (mother’s love), ‘*jee bharke pyar karna’* (love with all the heart); ‘maa ki jhappi’ (mother’s hug) and ‘*bander jaise lagana’ (*place on mother like monkeys do). ‘*Chaati se chipkana’ (*to stick to the chest) was felt to be most appropriate as it indicated that the baby’s skin would be in close contact with the mothers and did not indicate mere placing of the baby on the chest.

#### Household environment

For prolonged uninterrupted practice of KMC, several features within the home are important. These are availability of privacy, a toilet close to the mother’s room, ability to regulate temperature and humidity, availability of sufficient ventilation and adequate light for mother-baby interaction, and appropriate back rest. Three-fourths of the mothers had access to a toilet within the household close to their rooms, only a quarter of mothers needed to go to the field for defecation. Though half of the households had natural light during the day, it was insufficient as windows were always kept shut. Mothers’ rooms were dark requiring use of lights to be switched on even during the day. Rooms were poorly ventilated and hot and humid in summer. Using electric fans, coolers, watching television and listening to the radio were not recommended for recently delivered mothers, as these were believed to harm the mothers (who are considered to be weak post delivery) and their babies.

In the 6 weeks post delivery period, the usual practice was to place mother and baby on traditional cots with wooden frame, the *khaat or charpai (*bedstead consisting of a web of rope netting)*.* These cots are preferred in this setting as families feel that the flexibility of jute prevents backache as it takes the shape of the mother’s body. Besides, the jute can be easily washed to remove mother’s blood and baby’s urine and stools. Cots were often placed away from the wall to prevent evil spirits entering mother’s body. This meant that the mother did not have back support.

#### Key findings of the household trials

Twenty-eight mothers and 29 newborns (one set of twins) participated in the trials. The mean age of mothers was 24 years (SD 5). All deliveries, except one, were vaginal; one woman had undergone Caesarean section. All weighed less than 2500 g; 16 babies weighed < 2000 g. Two-thirds were facility births. The median age at which the team reached the mother at home was 40 h (range 2 to 167 h).

Around 60% of mothers said their baby was small. Of the two babies who were born preterm, both mothers said that the baby was born “before time”.

Of the 29 babies, 1 died and received 1 day of KMC; 3 were referred to hospital post enrolment- a set of twins who received KMC for 8 days, one received KMC for 2 days, 2 moved away from the study area- one received KMC for 3 days and another received for 13 days. Of those not lost to follow up, KMC was continued in all until 7, 21 or 28 days of life. The mean duration of practicing SSC was 5.9 h (SD 3.2) per day. Around half the babies wriggled out at around 3 weeks of age.

In the HH trials too, around two-thirds of mothers did not like the disposable diapers when offered to them and continued to use home-made cloth diapers. Ten mothers used the binders to hold the baby in place. Interestingly, contrary to what was reported during interviews, in a quarter of the families, fathers and grandmothers did SSC.

The common barriers while practicing KMC reported during HH trials, along with the solutions to overcome these, are presented in Table 1.

All babies were fed colostrum after birth. A fifth of mothers gave pre-lacteal feeds such as tea, sugar or glucose water. Breast feeding was initiated within an hour of birth by half of the mothers; four-fifths were exclusively breastfed at 28 days. Twenty-two of the 28 mothers breastfed their babies on demand; or 8–10 times during the day and 4 to 5 times at night. Three quarters said that they were able to feed the baby while still in the KMC position.

#### Factors motivating mothers to practice KMC

Mothers said that they liked doing KMC as their babies became healthy (25/28), gained weight (17/28), became active (16/28), fed better than before or fed more frequently (11/28) and suckled with greater strength (10/28). They also mentioned that their milk flow had increased. A quarter said their babies became beautiful (*maluk lagti hai, mashaallah shakal bahut sahi lagti hai!),* and the baby’s face glowed (*raunak aa gayi hai*). Two mothers said their babies became calm, slept peacefully and did not fall ill as often as other siblings. Four-fifths of the mothers said they would recommend KMC to others to enable them experience the immense benefits that they had observed.

#### Lessons for the design of the randomized controlled trial

The findings of the HH trials revealed that all mothers were willing to practice KMC when initiated at home once the benefits were explained to them and solutions devised to problems identified. KMC was practiced for several hours and benefits reported by all. Fathers and grandmothers supported mothers in doing KMC. Table 2 lists issues emerging from HH trials that provided inputs to the intervention package that was designed. Additionally, the barriers reported and solutions devised to overcome these (Table 1) would be used to develop a counselling guide for the RCT. The components of the final package for the RCT included initiation of KMC at home soon after birth, promotion and support for early, exclusive breastfeeding and continuous SSC till the baby is aged 28 days or wriggles out and no longer accepts KMC. Skilled higher level workers equivalent to government ANMs were thought to be more appropriate for helping mothers initiate KMC at home with subsequent follow up and problem solving being done by workers equivalent to government ASHAs. It was also felt that frequency of visits to the household could be reduced. For the RCT, we made the number of visits consistent with government guidelines with the addition of only two more visits [20].

**Table 2:** Lessons Emerging from Formative Research for the Randomized Controlled Trial

Issues	Role of formative Research in Shaping Intervention Package for the RCT
How long to practice KMC and for how many days	Scientific guidance on optimal duration of practicing KMC per day (in hours) and the total duration (in days) was lacking. We advised mothers to give KMC, preferably for 24 h in a day and continue giving till 28 days of infant age or till the baby wriggles out (whichever was earlier). Both these suggestions emerged feasible and acceptable in the formative research. These findings served as an input in the main RCT
Position of mother in SSC	Supine and semi-reclining positions were most comfortable and preferred. The same were advised in the main trial
Use of Binders	Women were not comfortable or confident moving around or doing household chores, with baby in SSC supported by binders, feared that baby will fall. Binders therefore, were decided to be provided to only those who ask for them
Use of Diapers	Diapers were considered unhygienic and thought to cause skin rash. Also, it was believed that they increase the gap between baby’s legs leading to deformity. Therefore, it was planned to offer only to those who asked for.
Personnel to deliver the intervention	SSC initiation and support for initial few days required skilled counselling, effective demonstration, persistent encouragement, empathy and problem solving ability to bring about behaviour change activation. ANM like workers, with higher education and skills were considered appropriate for initiation; ASHAs were able to support mothers during follow up visits
Frequency of visit	Frequent visits needed initially, could be reduced later. Substantial support and encouragement needed during first 3 to 4 days. Less frequent visits needed subsequently once the mother was comfortable doing KMC.
Weight cut off for enrolment	Babies with birth weight ≥ 1500 to ≤2250 g were planned to be included in the study, as babies weighing > 2250 g did not stay in SSC for more than 4 to 5 days (wriggled out earlier) and those weighing < 1500 g required hospital care.
Age cut off for enrolment	Very early enrolment would not be feasible for facility births. A window period of 72 h of birth was considered feasible to initiate KMC at home
Involvement of male members in providing KMC to the baby	Male family members participated enthusiastically in providing SSC; they were planned to be involved from the outset in the RCT

## Discussion

This is the first experience from India, and possibly the world, to develop an intervention package to initiate KMC at home, using formative research including household trials. The development of the package took into account cultural beliefs, traditions and inputs from families to identify solutions to promote and support practices for a new intervention.

Our study indicates that recognition of small babies by appearance and their need for special care was prevalent in the community. Kangaroos were not recognized and KMC and its benefits were known by very few. In spite of concerns about feasibility, there was agreement that awareness of benefits would lead to high adoption rates of KMC. Fatigue, weakness, backache post delivery, mothers’ poor health and lack of family support in nuclear families were identified as barriers. Enablers were the cultural practice of postpartum rest and family support in extended families. The observed improvement in baby’s health seemed to be key motivators for adoption. Mothers were happy that their babies gained weight, looked healthy and beautiful, fed better, and became active and fell ill less frequently compared to their experience with previous LBW babies.

The study identified several suboptimal newborn caring practices such as giving of pre-lacteal feeds, bathing soon after birth and formula feeding. Introducing KMC for LBW babies may help displace such practices and positively influence home care practices.

Household trials provided a way to practically demonstrate to mothers the experience and practice of a new intervention and feel its benefits. They provided a more reliable assessment of the feasibility of intervention adoption. Several barriers and facilitators were identified, some of them reported by previously published studies [21, 22]. The key barriers were lack of awareness of KMC among mothers, lack of help with KMC practice, fear of hurting infant, and mothers’ pain and fatigue post delivery. Motivators included increased mother-infant attachment, feeling of confidence, satisfaction in seeing the baby grow and family support. Positive perceptions of health workers have also been reported by others [18]. Our study showed that repeated dialogue with the mother and family members together with practical support can boost adoption rates. In our conceptual model, substantial emphasis was placed on social acceptance and community endorsement of KMC as a mediator for adoption. However, during the HH trials, it was evident that individual counselling at family level along with support by study workers, in the absence of significant negative societal perceptions, was sufficient to result in high uptake of KMC. We also learned that fathers can play a significant role in KMC adoption and continued practice. Although mothers’ and families’ motivation and skills could be mobilized for adoption of KMC, the high adoption rates also reflect the intensity of good quality counselling and support. Programmes to support home-initiated KMC will require similar level of skilled workers and intensity of delivery to succeed.

Additionally, solutions to barriers when evolved through a participatory process involving the mothers and other family members and within the domains of their age old socio-cultural and traditional belief system were acceptable and sustainable.

The major limitations of the study were inability to test early initiation of KMC at home due to the high proportion of facility births. In absence of an objective measure of SSC duration, compliance to KMC was ascertained through mothers report, although surprise checks were made at home to reconfirm reported behaviour.

## Conclusions

cKMC is relevant for LMICs where home deliveries still occur, and babies born in facilities are discharged early. Formative research with household trials not only ascertained feasibility and acceptability of a relatively new intervention and its adoption, it also helped design the intervention package for a large randomised controlled trial aimed to ascertain mortality impact. High adoption rates can be achieved but require intense and skilled counselling with support. The government ASHAs and ANMs can be trained to initiate and support mothers at home to practice KMC. Considering that about 5% of all births are less than 2000 g, it seems feasible to implement KMC within the existing health system.

## Abbreviations

ANM: Auxiliary Nurse Midwife
ASHA: Accredited Social Health Activists
cKMC: community-initiated kangaroo mother care
FGD: Focus group discussions
HH: Household
IDI: In-depth interview
KMC: Kangaroo Mother Care
LBW: Low birth weight
LMIC: Low and middle income countries
RCT: Randomized controlled trial
SSC: Skin-to-skin contact
